# Toward emotional mediation: generative AI in art therapy for psychosocial health support

**DOI:** 10.3389/fpubh.2025.1690119

**Published:** 2025-11-03

**Authors:** Zhongyu Shi, Danqing Yin

**Affiliations:** ^1^Shanghai Film Academy, Shanghai University, Shanghai, China; ^2^School of Biomedical Sciences, Li Ka Shing Faculty of Medicine, The University of Hong Kong, Hong Kong SAR, China

**Keywords:** AI-based art therapy, emotional mediation, human–AI interaction, affective role of AI, explainable AI, therapeutic co-creation, digital mental health

## 1 Introduction

The American Art Therapy Association defines art therapy as “a regulated mental health profession that uses active art-making, the creative process, applied psychological theory, and human experience within a psychotherapeutic relationship” (1). Similarly, the British Association of Art Therapists defines it as “a form of psychotherapy that uses art media as its primary mode of communication” (2). Since the 1960s−70s, art therapy has been recognized as promoting health and well-being (3, 4). As a psychological intervention, it emphasizes emotional externalization via visual expression and fosters patient–therapist emotional bonds, providing a key non-verbal communication method (5, 6). Today, art therapy encompasses various media such as painting, sculpture, photography and etc.

The development of artificial intelligence (AI) for image generation, language interaction, and pattern recognition has introduced new possibilities for art therapy. Some scholars argue that AI systems are creative, with the capacity to generate novelty (7). Moreover, AI can serve as a source of inspiration and a practical tool for those with little artistic experience (8). Interactive AI systems offer real-time feedback, stimulating users' imagination and modes of expression (9). For example, collaborative tools like Coco Sketch, GauGAN, and SmartPaint, along with AI-enabled apps like Mind Palette, combine generative AI with therapeutic goals to support mental health (10–13).

## 2 Trajectories and practical innovations: AI-driven art therapy

Current perspectives in the literature suggest that AI use in art therapy is moving from media-based support toward more structured forms of intervention. An integrative review of the role of AI in art therapy conceptualizes AI as an expressive tool focused on creative collaboration, emotional externalization, and symbolic generation (14). For example, a small-scale study employed autonomous painting robots to co-create with users under a Diagnostic Drawing Series (DDS) framework, proposing the potential for an affective human–machine interactions (15). There are also co-creation platforms aimed at reducing technical barriers, wherein therapists have contributed to collaborative strategy design (10). Additionally, recent studies have begun to explore how AI might function in therapeutic contexts, with roles ranging from supportive tools to potential co-creators or semi-autonomous agents (14, 16). In one pilot study, AI-generated imagery was situated within the expressive therapy continuum (also known as ETC), demonstrating the possible use of AI to activate creativity across cognitive, emotional, and sociocultural dimensions (16). Following this trend, a further study showed that AI-enhanced drawing games improved engagement and cognitive stimulation in older adults with mild cognitive impairment. However, these activities were not conducted within a formal art therapy setting and did not appear to incorporate a therapeutic relationship facilitated by a trained art therapist, which is often considered a key element in art therapy (17).

Second, several emerging studies have explored the potential of AI as an assistive system in tasks such as assessment, emotional recognition, and therapeutic feedback. For instance, AI-assisted systems can support large-scale drawing evaluation and improve interpretability and diagnostic accuracy (18, 19). Meanwhile, tools such as TherAIssist allow therapists to use AI to collaborate on procedures related to feedback, tracking, and homework (20). In education, AI has helped with the analysis of emotional expressions in drawings by students with autism (21). Another study shows the capacity of AI to identify emotional cues and guide therapeutic dialogues, aiding patients with post-traumatic stress disorder in emotional release (22). Deep neural networks were used to integrate statistical and qualitative drawing evaluations (19), and reviews of AI interventions (i.e., from drawing agents to chatbots) have highlighted both their innovation and emerging ethical concerns and trust-building needs (17).

AI's role in art therapy might be expanding beyond expressive support to emotion recognition, feedback modulation, and spatial construction. As AI systems increasingly engage in tasks that involve affective perception, symbolic expression, adaptive regulation, and reflective prompting, their role begins to resemble therapeutic dynamics such as attunement and alliance, positioning AI as a potential emotional mediator in art therapy (23). Nevertheless, the current literature continues to focus more on discrete functional outputs (e.g., image generation and technical efficiency), and less on the processual and relational dimensions of emotion in AI outputs (24). Building upon prior notions of expressive vehicles, emotional resonance, and nurturing systems (14), emotional mediation emphasizes AI's emerging capacity to intermediate between internal states and symbolic articulation (25), offering a dynamic interface for therapeutic engagement.

This raises two central questions: can AI become an active modulator of emotional processes and not just an analytical tool? Will it be able to help build emotional connections and support healing in the emotionally charged settings of art therapy? These two questions then lead to the research question of this opinion study: can AI function as a new emotional mediator rather than merely a technical aid? In this study, we use the term “emotional mediation” not as a fixed clinical construct, but as a bridging concept to emphasize AI's emerging capacity to intermediate between internal states and symbolic articulation.

## 3 Advantages and limitations

### 3.1 Advantages

AI use in art therapy has been shifting from technical purposes to emotion recognition, expression, regulation, and connection (15, 26). Recent applications have suggested five potential functional mechanisms through which AI may support emotional intervention (27). First, AI serves as an emotional recognizer during early therapy. Facial expressions, vocal tones, and visual features (e.g., color or composition) can be analyzed to generate emotional profiles or mood trajectories, which may help in treatment planning. This ability to read and respond to affective cues parallels therapeutic attunement, in which the public health professionals aligns with the patient's emotional state. The AlphaDAPR system, for instance, uses interpretable image recognition to detect distress patterns (18), and image-based cues can be analyzed by AI to inform post-traumatic stress disorder treatment (15, 22).

Second, AI acts as an emotional externalizer by transforming implicit feelings into visualizations. This benefits people with limited verbal abilities such as children, trauma survivors, or individuals with autism (25). Specifically, prompt-to-image tools help produce symbolic and emotionally charged artworks, with tools such as Deep Think, guiding users into reflection via visual creation (10), and AI-generated cards stimulating emotional dialogue (26). These methods help translate unconscious emotions into processable visual symbols.

Third, AI functions as an emotional regulator, offering adaptive support through real-time feedback. When users experience distress during creation, AI adjusts its interaction style (e.g., pacing, prompts, or emotional tone) to support emotional stabilization (28, 29). In TherAIssist, AI tailors drawing tasks to sustain engagement (20), and Stable Diffusion studies have shown that AI-generated images can yield short-term emotional improvement (22).

Fourth, AI can serve as an empathic mirror, prompting deeper reflection and helping disadvantaged patients confront repressed feelings. In conditions such as post-intensive care syndrome (PICS), trauma-related recall may be triggered by AI-supported reflective systems (30), while other platforms combine affect recognition with cognitive-behavioral therapy-informed prompts to facilitate structured emotional awareness (14). These interactions mark a shift from AI as a passive assistant to a reflective agent—one that engages users in ongoing emotional dialogue. In this manner, these systems emulate elements of the therapeutic alliance (31, 32), particularly in their efforts to foster emotional trust, responsiveness, and collaborative engagement with users. Some researchers even envision AI serving as an “auxiliary therapist” capable of contributing independently to therapeutic processes (27).

Finally, AI systems may be used to help create emotionally supportive environments for users with anxiety or cognitive impairments, creating immersive, affectively tailored virtual spaces that offer containment, safety, and therapeutic “sanctuaries.” Indeed, virtual reality-based systems improve emotional stability in dementia care (33), anxiety and depression (34) as further demonstrated by XAIA, an AI-guided calming space for personal reflection and emotional sharing. The sense of presence in immersive environments is closely linked to the creation of emotions because these environments can evoke users' emotions through emotional transfer. This makes them powerful tools for influencing human behavior and facilitating therapeutic change (35).

Stretching from recognition to regulation, resonance, and space-building, the emotional role of AI in art therapy reflects a multidimensional progression, which is emerging through tools and emotional mediators that combine affective sensitivity with technological intelligence.

### 3.2 Limitations

While recent research has expanded AI in art therapy, several critical limitations have surfaced regarding the emotional domain and ethical–technical boundaries of AI integration. First, although AI-generated suggestions (e.g., mood-driven imagery) may support expression, they may override users' agency. When AI systems actively shape visual content, they risk diminishing patients' sense of ownership and self-awareness during therapy (14, 36). Biased or incomplete training datasets can distort emotional assessments, misrepresent symptoms, or oversimplify mental health conditions, particularly in diverse populations. In suggestion-heavy interactions, algorithmic defaults may even subtly steer emotional output toward “system expectations” rather than authentic inner states (10, 37).

Second, AI systems often process sensitive psychological cues such as facial expressions, vocal tones, drawing features, and emotion tags, raising serious concerns regarding surveillance and emotional transparency (24, 38). Continuous monitoring can also lead to anxiety, hypervigilance, or loss of privacy, as users may feel reduced to datasets rather than complex emotional beings.

Third, digital images can be easily stored, duplicated, and circulated, and cause distress if they resurface unexpectedly. Conversely, although physical artwork is more difficult to preserve, its spatial and temporal presence may serve as a lasting reminder of past trauma, possibly triggering shame or discomfort (15). Both outcomes highlight the emotional importance of therapeutic content and the need to carefully manage its storage and access.

Accordingly, we must recalibrate human–AI dynamics in emotionally sensitive contexts. While AI promises to enhance emotional expression and connection, its interventional nature requires a greater awareness of power dynamics, informed consent, and emotional boundaries among stakeholders. As technology becomes embedded in the therapeutic process, it is crucial to balance between assistance and empowerment, as their imbalance may increase the risks of AI shifting from a supportive presence to a directive force (39).

## 4 Discussion

**Figure 1:**
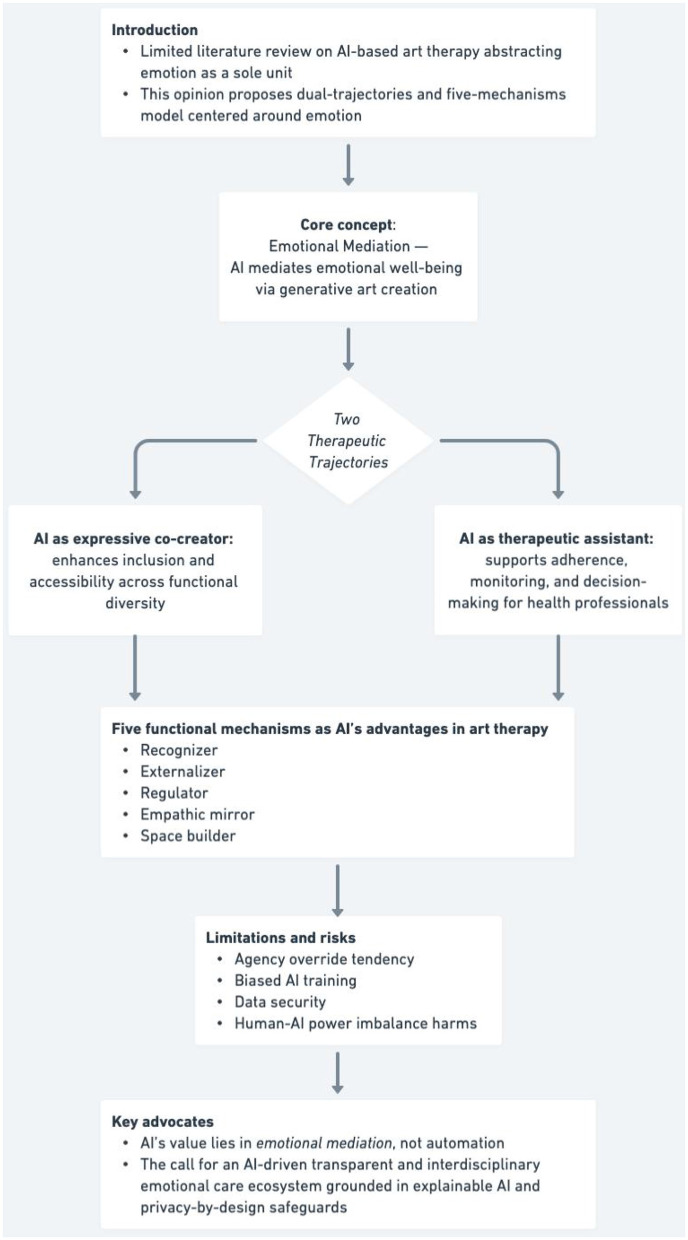
Graphical abstract summarizing key points.

This paper examines how AI is evolving from a technical tool into a system that can support therapeutic processes by recognizing, responding to, and facilitating emotional expression in art therapy. In contrast to existing studies that contextualize AI-based art therapy primarily as psychotherapeutic tool, this perspective repositions generative AI's role in emotion mediation (40, 41), examing AI-driven art therapy in the lens of emotion. We proposes a conceptually innovative dual-trajectory framework supported by a five-mechanism model centered around emotion—encompassing the emotional recognizer, externalizer, regulator, empathic mirror, and space-builder—framed through public health and psychosocial context. Particular attention is given to emotional–ethical boundaries of human–AI interaction (Figure 1).

While not a replacement for human therapists, AI systems increasingly emulate specific functions—such as recognition, attunement, and expression facilitation. To realize AI's potential in these domains, future research and practice must develop an AI-driven psychosocial care ecosystem bridging technological rationality and human-centered compassion. A foundational step involves implementing explainable AI methods to enhance transparency and therapeutic trust. Techniques such as Shapley additive explanations (SHAP) and local interpretable model-agnostic explanations (LIME) are used to visualize how input features influence AI decisions, allowing therapists to trust AI-supported outcomes (38, 42). By showing therapists how AI interprets emotional inputs, these tools support accountability and patient confidence in AI-supported outcomes (43).

However, this remains a nascent area of research. Most abovementioned studies are exploratory, often based on small-scale or preliminary frameworks. As such, these findings should be interpreted as early contributions that highlight potential trajectories rather than established evidence.

Equally important is the development of collaborative approaches involving professionals from different fields. Art therapists, public health experts, social workers, AI engineers, designers, and ethicists should work together to explore how AI might support emotional expression and psychological care. Integrating multiple types of input—such as facial expressions, drawings, and written language—could improve how AI systems respond to users' emotional states (44). Open testing environments, such as pilot programs or controlled digital platforms, could show researchers how AI interacts with users in emotionally sensitive settings (45).

In summary, AI-enabled art therapy has the potential to transform digital mental health practices, complementing traditional face-to-face therapy by offering additional channels for communication and engagement, especially for people with cognitive or emotional challenges (46, 47). Rather than replacing human therapists, AI can serve as a vital emotional mediator in psychosocial health care.
